# Advancing “Autonomous” sensing and prediction of the subsurface environment: a review and exploration of the challenges for soil and groundwater contamination

**DOI:** 10.1007/s11356-022-25125-8

**Published:** 2023-01-13

**Authors:** Greg B. Davis, John L. Rayner, Michael J. Donn

**Affiliations:** CSIRO Land and Water, 147 Underwood Avenue, Floreat, Western Australia 6014 Australia

**Keywords:** Contamination, Sensor, Automation, Groundwater, Soil, Digital twin

## Abstract

Can we hope for autonomous (self-contained in situ) sensing of subsurface soil and groundwater pollutants to satisfy relevant regulatory criteria? Global advances in sensors, communications, digital technologies, and computational capacity offer this potential. Here we review past efforts to advance subsurface investigation techniques and technologies, and computational efforts to create a digital twin (representation) of subsurface processes. In the context of the potential to link measurement and sensing to a digital twin computation platform, we outline five criteria that might make it possible. Significant advances in sensors based on passive measurement devices are proposed. As an example of what might be achievable, using the five criteria, we describe the deployment of online real-time sensors and simulations for a case study of a petroleum site where natural source zone depletion (NSZD) is underway as a potential biodegradation management option, and where a high-quality conceptual site model is available. Multiple sensors targeting parameters (major gases and temperature influenced by soil moisture) relevant to the subsurface NSZD biodegradation processes are shown to offer the potential to map subsurface processes spatially and temporally and provide continuous estimates of degradation rates for management decisions, constrained by a computational platform of the key processes. Current limitations and gaps in technologies and knowledge are highlighted specific to the case study. More generally, additional key advances required to achieve autonomous sensing of subsurface soil and groundwater pollutants are outlined.

## Introduction

We live in a digital, tracked, and sensorized world (Gunn 2020). Global positioning systems (GPS) locate us and our destinations, sensors regulate our household temperatures, and we can ask web-assistants to find any answer to questions that can be found on the internet. Humanity made it to the moon and back over 50 years ago and can automatically land aeroplanes (hands free). However, in relative terms, representing, measuring and predicting the natural environment remains elusive.

This is especially the case for subsurface environments where degraded and polluted soils and groundwater threaten ecological systems, human health and water and soil quality and health (Li et al. 2021), and accelerated efforts for perhaps 40 years since Love Canal (Phillips et al. 2007) triggered massive research and investigative efforts in the United States of America, and other countries worldwide. The cost globally to manage and assess such contamination is many billions of dollars annually. For nine countries in Europe, not including Germany nor the UK, the estimate was 1.4 billion euros per annum in 2010–11 (van Liedekerke et al. 2014).

While we fly drones and aeroplanes and use satellites deploying geophysical techniques to map vegetation and landscapes (Roy et al. 2017; Liang et al. 2020), much below a depth of a few centimetres from the ground surface, automated mapping and modelling is challenged. Ground-based geophysics has been a staple of mineral exploration for broad-acre mapping, targeting orebodies or structures that would direct mining and oil and gas exploitation. Adequate definition of shallow soil/groundwater targets using geophysics is elusive, especially for low pollutant concentrations (e.g., per- and polyfluoroalkyl substances—PFAS) that pose significant risks (Brusseau et al. 2020; Rayner et al. 2022). Where contamination occurs, assessing and predicting the environmental condition of soils and groundwater remains laborious. Investigations at sites are staged, not automated, laboratory analysis of samples take days to weeks, data integration and reporting is months apart and computational platforms needed to integrate such data struggle to provide predictions of contaminant migration and distributions that are realistic and rapid let alone providing near real time outcomes that are updated and linked to transient environmental data.

Digital twins are increasingly used as computational representations of key system processes linked to information from sensors to quantitively assess and manage systems, many in real time (Gelernter 1991). As computational representations of closely engineered systems (e.g., automotive engines, chemical production) digital twins are increasingly real, and meshed with robotic and automated systems (Grieves 2019). We can now predict aspects of climate reasonably well (Sillmann et al. 2017), and increasingly aspects of landscape function (Wang et al. 2021). In agriculture major advances have been achieved in linking sensors through the Internet of Things (IOT) and associated communication and informatics to landscape and soil function and productivity (Mehmood et al. 2022). However, despite enormous advances in sensors, measurement technologies, and computational and predictive power, sensing and predicting the pollution status and environmental condition of subsurface soils and groundwater remains fraught.

**Fig. 1:**
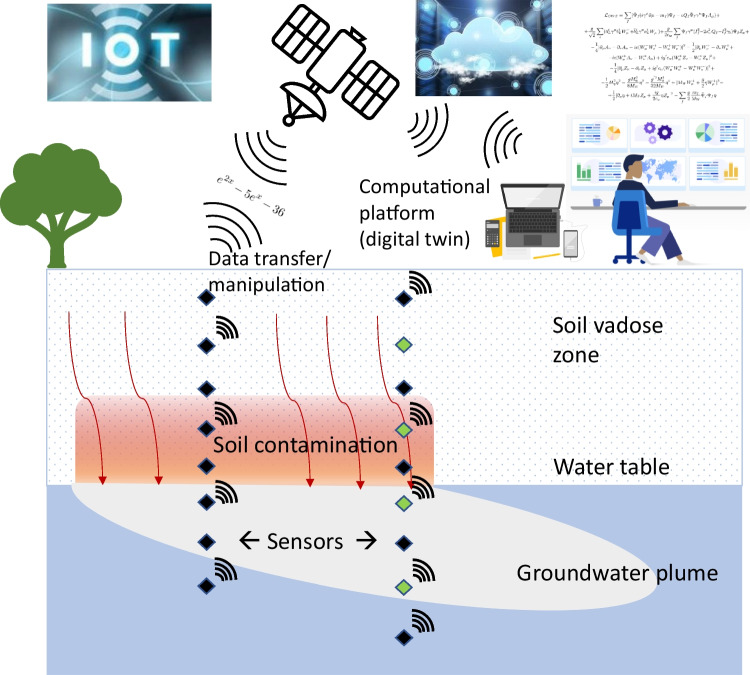
Schematic showing an ambitious depiction of a linked future system of “autonomous” sensors (green and black diamonds) deployed into the subsurface to monitor pollution distributions and processes with data wirelessly telemetered (black radial arcs) and linked to the internet of things (IOT), an intelligent database and computational platforms to track and interpret subsurface conditions and allow real time management responses

Here we overview historical efforts to accelerate reliable soil/groundwater measurements and to develop more rapid approaches and technologies for tracking contaminants in the shallow regolith. We include a brief assessment of computational advances that may link with measurements and data. We explore steps to advance technologies to move towards a true digital twin of soil and groundwater environments, with computational platforms that self-adjust in response to “autonomous” sensor networks—a step towards automated reporting and decision making (Fig. 1). A pipe dream or a possibility? Finally, we provide a field example of advancing such sensing and interpretation linkages that perhaps shows a glimpse of what may be possible, when we know ‘enough’ about the fundamental properties and processes governing contaminant risks and are able to measure them and represent them virtually.

We note that “autonomous” is often taken to mean automatic sensing in a moving device such as a driverless motor vehicle, or remote submarine probe. Here for subsurface environments, we are using the term to mean self-contained, and perhaps intelligent sensors with some interpretive capacity, rather than mobile. Such sensor systems might provide alerts when regulatory criteria are exceeded or may automatically adjust the frequency of sensing intervals to better resolve a concentration peak.

## Framing where we are at

Many guidelines, standards, and frameworks exist describing measurement and characterization approaches for contaminants in the subsurface environment. For assessing site contamination, Australia has a national environment protection measure (NEPM) that was last updated in 2013 (NEPM 2013). It describes expected approaches and links to Australian sampling and assessment standards and guidelines. In the USA, the US EPA has a broad suite of methods and approaches that cover most site investigation approaches (CLU-IN 2022). Europe and many other countries have similar guidelines and developments (Theocharopoulos et al. 2001; UK EA 2022). While all might promote technology advancement, automated on-line sensing and reporting with linkages to digital and computational platforms are lacking.

### Measurement of what?

To establish a valid conceptual site model of contaminant behavior, it is essential to measure aspects of the physical environment that the contaminant is residing in, such as soil texture, aquifer stratigraphy and properties, and perhaps other chemical transport parameters such as soil moisture, thermal properties, and gas diffusivities. These all govern how the contaminant may behave within the system as a whole. Surface and downhole geophysics along with drone or satellite deployed sensors can typically assist with mapping structures and in some cases soil moisture and textural changes (Roy et al. 2017; Kelleher et al. 2018). Here however, we focus on phases and techniques that target the contaminant of interest or associated chemical parameters.

To assess contaminant risks, sampling of the subsurface targets investigation and measurement of contaminants residing in (i) soil and aquifer sediments, (ii) soil porewaters, (iii) groundwater and/or (iv) soil gas. Depending on the property of contaminants (e.g., their solubility, volatility, density, partitioning behaviours) sampling these phases can be indicators, or definitional, of contaminant distributions and risk in the subsurface.

Soil and aquifer sediments: Historically, soil and geological samples were recovered to assess if soil was suitable for agriculture or if orebodies or oil/gas reservoirs were minable. Beyond such applications, drilling, and preservation and handling of such samples were improved as the need arose to assess the chemical/pollution status of subsurface soils and aquifer sediments (Theocharopoulos et al. 2001). Percussive cable tool techniques gave way to rotary hollow stem augers, diamond drilling, sonic rigs, push-rod geoprobe rigs and other methods and techniques that would preserve soil core structure and chemistry (The Driller 2022). In some cases, freezing of cores was proposed in unconsolidated formations to recover “non-disturbed” in situ soil core samples (Sego et al. 1994).

Soil porewaters: Soil porewater chemistry has been of keen interest for some time in agricultural practices, especially for optimising nutrient, pesticide and fertilizer applications (Wagner 1962). It has been less used for regulation or routine tracking of contaminant risks. Given the soil vadose zone is often a pathway for contaminants to be transported into groundwater, there is increasing interest in promoting soil-porewater as a sentinel measurement prior to groundwater impacts (Dahan 2020). Lysimeters in various forms have been developed and their use standardized for sampling soil porewaters in situ (ASTM 2018). They have been adapted and evaluated for assessing the relative mobility of contaminants leaching through soils (Patterson et al. 2000a) and promoted for use in evaluating recent threats from PFAS (Anderson 2021). However, as pointed out by Davis and Rayner (2021) site characterization guidance is less developed for lysimeters especially in defining spatial deployment or frequency of sampling compared to guidance available for groundwater sampling or soil coring approaches.

Groundwater: For groundwater, historically, boreholes and piezometers were emplaced to pump water and measure heads and water levels in aquifers. This transitioned toward representative sampling of boreholes to gain samples for chemical and pollutant measurement and groundwater plume mapping. Prior to recovery of “representative” groundwater samples, purging of borehole stagnant casing storage from short-screened boreholes was promoted (Barber and Davis 1987), but more commonly adopted now is low-flow sampling which was introduced to avoid water column and colloidal disturbance and to minimize handling of contaminated water volumes during purging (Puls and Barcelona 1996). To better define risk and plume movement sampling from long screened boreholes moved to discrete interval multi-depth sampling to define pollution plume depth distributions (e.g., Ronen et al. 1986; Davis et al. 1993). Such multi-depth sampling often demanded development of new analytical techniques to be able to utilize smaller groundwater sample sizes especially if depth interval spacings were narrow or fluid recovery was restricted (e.g., Patterson et al. 1993; Jawitz et al. 2002).

Soil gas: Gases in shallow soils have been deemed critical for agricultural purposes (Gliński and Stępniewski 1985) but gained importance as a shallow survey tool to map subsurface volatile organic compounds (VOCs) as a measure of subsurface contamination in soil and groundwater (Marrin and Kerfoot 1988; Barber et al. 1990a; Davis et al. 1991) and for contaminant vapour risks (Davis et al. 2021). Recently, soil gas sampling has become a strong focus for quantifying the rate of natural source zone depletion of petroleum light nonaqueous phase liquids (LNAPLs) in the subsurface (Sookhak Lari et al. 2017; Davis et al. 2022), since VOC and oxygen consumption, and methane and carbon dioxide production are strong indicators of LNAPL biodegradation processes and rates (Garg et al. 2017; Sookhak Lari et al. 2019).

Such soil and groundwater sampling advances provide us with standardized approaches and usually representative samples that aligned with or modified our site conceptualizations. However, in nearly all cases sampling disturbs the subsurface or provides a disturbed sample (as in soil recovery) and for groundwater, lysimeter porewater, and gas samples all are largely volume-averaged concentrations depending on the duration and volume recovered in a sampling event. Because of this, ambitions arose to perhaps measure concentrations in situ in the subsurface, without having to recover samples or undertake laboratory analysis. A number of techniques and approaches have been advanced to seek to address this.

### Shallow geophysics and other techniques

Geophysical techniques initially used in the petroleum and mining industry were adapted to target and map shallow contaminant features in soils and groundwater. The techniques relied on being able to distinguish the target zone from the bulk soil/aquifer often related to boundaries that are relatively well defined. Binley et al. (2010) outlined the opportunities and challenges of using hydrogeophysics for characterization subsurface features relevant to soil and groundwater. They focused on controls on water movement for emerging applications such as hyporheic zone processes and soil–water-plant interactions. For contaminant mapping, Buselli et al. (1990, 1992) used transient electromagnetics and direct current (DC) resistivity to map salinity and the development of leachate plumes from waste landfills. Using ground penetrating radar, Hwang et al. (2008) tracked the mobility of a tetrachloroethene spill release into a sand aquifer over time. Such surface techniques enabled measurement of gross plume and contaminant features such as high electrical conductivity contrast plumes, NAPL presence in a formation, and possibly biodegradation signals (Atekwana and Atekwana 2009). Mapping of low concentrations that may still pose a risk (e.g., benzene, PFAS) eludes such techniques.

Geoprobe techniques have been developed to partner downhole geophysics during drilling with additional chemical sensing. Examples of the latter are laser induced fluorescence (LIF) for detecting petroleum hydrocarbons (García-Rincón et al. 2020) and membrane interface probes to map vertical distributions of VOCs such as chlorinated or petroleum hydrocarbons, and often linked to onboard chemical detectors (Christy 1996). This allows spatially dense vertical measurement of both soil strata properties and volatile contaminant concentrations and enables rapid in-field decisions as to the next-best locations and depths to investigate.

### “Passive” monitoring

To avoid sample disturbance, beyond geophysics, techniques were developed to measure concentrations and processes in situ in the subsurface. Often this involved passive measurement techniques whereby a device that was placed in soil or groundwater would consist of a trapping medium (e.g., activated carbon, solvent, resins, water, gas) or sensor device relevant to the chemical pollutant of interest (e.g., organic or inorganic compounds). Many devices were based on diffusion through a well-defined diffusion barrier or membrane. A review and timeline of development from early air monitoring devices to more recent water devices were reported by Namiesnik et al. (2005). Some sampled and measured concentrations, but others measured fluxes, the latter being an indicator of mass loading to the environment.

For inorganics in groundwater, Ronen et al. (1986) developed membrane-covered, water-filled vials termed dialysis cells, which could be deployed into drilled boreholes to study depth profiles of hydrochemistry in groundwater. The membrane allowed ion exchange until chemical equilibrium was achieved. After being left for a period, the string of dialysis cells would be recovered from the well and samples analyzed. Laor et al. (2003) expanded this technique for measurements of VOCs. Soedergren (1987) used hexane filled dialysis membranes to understand pollutant uptake into organisms, and Berho et al. (2013) adapted this approach to measure pesticide concentrations in groundwater. Pinasseau et al. (2020) promoted a disk-based sampler placed within groundwater bores for measuring polar and semi-polar compounds such as atrazine. A range of soil and groundwater passive techniques were promoted and further developed for use with chlorinated compounds in urban redevelopment projects in Europe (CityChlor 2013). There have also been recent advances for PFAS passive samplers using specialist modified sorbents (Hale et al. 2021; Hartmann et al. 2021).

Little has been done to link such passive sampling techniques to sensors, detectors or online reporting. Many standalone sensors for chemicals of interest are actively being developed. For example, a recent review of PFAS sensors spanned a wide range of possible approaches (e.g., dyes, colorimetric, fluorescent, nanoparticles) in response to the need for “fast, inexpensive, robust, and portable methods to detect PFAS in the field” (Menger et al. 2021) but found that many challenges remain. Linking such detectors in a robust way to passive sampling systems for automated reporting would be a major advancement.

To measure in situ fluxes of groundwater and contaminants, Annable et al. (2005) developed passive flux meters (PFMs), which consisted of sorptive material containing a tracer and lowered into a well. After a set period estimated from approximation of local groundwater flow rates, the PFMs were recovered from the well and analysis of the sorbent for the remaining mass of tracer and the contaminant in groundwater that had adsorbed to the PFM gave estimates of water and chemical fluxes in groundwater. These were verified in the field and used in combination with other concentration and mass flux measurement techniques (Basu et al. 2009). Alternative time-integrated “flux” methods have also been developed (Martin et al. 2003). While a passive and valuable method, these time-integrating devices require installation and recovery, along with laboratory extraction and chemical analysis, making the measurement technique somewhat labour intensive and lacking in terms of real-time reporting.

Recognizing the importance of measuring gases (e.g., oxygen, carbon dioxide, methane) and VOCs for groundwater quality and health, increasing efforts were focused on characterising soil gas composition and exchanges with underlying groundwater across the capillary fringe and with the atmosphere (Rivett et al. 2011; Ha et al. 2014). To provide vertically-continuous depth profiles across the capillary zone and under water saturated or unsaturated conditions, Barber and Briegel (1987) developed the diffusion cell technique; a polymer membrane tube that when buried in the subsurface would yield an equilibrated gas within the interior of the tube that could be sampled and analyzed for gases/VOCs. These were deployed to measure oxygen concentrations under seasonal water table variations with active tree-root respiration (Barber et al. 1990b), and at landfills to measure methane transport from groundwater leachate plumes below the water table up through the groundwater and soil profile (Barber et al. 1990a). These were also deployed to measure VOCs such as a trichloroethene plume (Benker et al. 1997) where the depth to the water table was at the limit of suction to be able to recover groundwater samples from multilevel samplers (i.e., theoretically a 10 m depth).

The technique was automated by connecting diffusion cells to appropriate sensors. For VOC probes functionality and testing are described by Patterson et al. (2000b), for oxygen probes by Patterson and Davis (2008), and for carbon dioxide probes by Patterson et al. (2013). They were deployed and tested for measurement of bioreactive subsurface systems (pyrite oxidation by Patterson et al. 2006, biodegradation of chlorinated hydrocarbons by Davis et al. 2009a), for monitoring remediation efforts (Johnston et al. 1998), and in combination with alternate concentration and mass flux measurement techniques to characterize a trichloroethene plume in groundwater (Basu et al. 2009). Functionally, the oxygen probes (diffusion cell and electrochemical sensor) were typically buried with a wire access line to a datalogger and telemetry, but the VOC and carbon dioxide probes required more surface infrastructure to circulate a gas stream through the diffusion cells to an appropriate detector at ground surface, linked to a datalogger and telemetry.

### Computational advances—towards a “Digital Twin”

A conceptual site model (CSM) encapsulates the essential features necessary to include within a modelling framework. The subsurface encompasses variable water-saturated zones and variations over seasons and precipitation events, many phases (air, water, soil, biological and potentially NAPL) with relative fractions and variations, and soil and aquifer hydrophysical, stratigraphical and biogeochemical attributes. Sources of contamination into the subsurface can be multiple and may be at the ground surface or at some depth, for example from underground storage tanks or leakages from buried pipelines (Sookhak Lari et al. 2016). To add to the complexity, the properties of a contaminant (solubility, volatility, sorption potential, degradability), interacting with such attributes of the subsurface make computational representation challenging.

Computational representation of the subsurface has advanced significantly over the last 30 years, along with computational speed and capacity. Establishing water flows is critical for estimating the movement of soluble contaminant species (Hutson and Wagenet 1995), and where petroleum or solvent NAPLs are spilled, multiphase flows need to be considered (Putzlocher et al. 2006; Johnston and Trefry 2009). For multicomponent NAPLs such as petroleum fuels, tracking of multiple fluid phases (air, water, NAPL) as well as partitioning components that may pose risks (e.g., benzene in fuels) needs to be undertaken concurrently (Sookhak Lari et al. 2016).

Where components also biodegrade, microbial populations and electron acceptor and donor chemicals need to be also embedded in the modelling platform. For groundwater, Prommer et al. (1999) was one of the first to link groundwater flows to multicomponent chemically-balanced biogeochemical reactions, and applied it to quantify the natural attenuation of a petroleum fuel plume in a sandy aquifer. Clement et al. (2004) linked key modules for dissolution, sorption and biodegradation reactions to simulate dense chlorinated NAPL (DNAPL) behaviour in groundwater. Recently, Engelmann et al. (2021) evaluated the robustness of DNAPL source zone representations against available data. Sookhak Lari et al. (2022) coupled all key multiphase, multicomponent and biogeochemical processes to progress the creation of a two-dimensional digital twin of natural source zone depletion (NSZD), by applying it to the crude oil pipeline leak in Bemidji, USA. The paper showed the potential to capture all key processes within such a computational modelling platform. However, in no way was the model linked to data from the site (apart from validating the model itself) and did not consider the third dimension. The model typically took 30 days to run a simulation over 30 years; three-dimensional modelling was estimated as being 4–5 times slower (Sookhak Lari—personal communication). Modelling in three dimensions may not always be required.

### Efforts to accelerate site investigations and monitoring

Since Love Canal over 40 years ago in 1978–1980 which led to the creation of the US Superfund Program (Comprehensive Environmental Response, Compensation & Liability Act (CERCLA) under US President Jimmy Carter) (Phillips et al. 2007), there has been an urgency to systematically assess the risks of contaminants in the subsurface and particularly in groundwater. Developments of in-field and other techniques have continued since that time to ensure high quality data, risk assessments and management options. However, as indicated, investigations can be prolonged and expensive enjoining efforts to accelerate investigations.

Accelerated site characterization (ASC) and advancing of measurement techniques and tools have been described above, inclusive of downhole geophysics and investigation tools. In the nineties, many saw the need to expedite site investigations (Burton et al. 1993; Robbat 1997) and these were advanced via standards and guidance (ASTM 1998, US EPA 1997). As early as 1994, Diran and Phillips (1994) sought to accelerate contaminant delineation promoting an interactive soil gas survey technique for vadose zone plume delineation, by on-site gas sample analysis. Interestingly, in Australia when techniques for characterising sites were reviewed in 2006 it was noted that “No implementation of ASC techniques is apparent in Australia, despite savings estimates of up to 40–50% for some applications in the United States of America” (Davis et al. 2006). Downhole geoprobes and other ASC techniques are now commonly employed globally (e.g., García-Rincón et al. 2020). The US EPA was a leader in supporting the development and testing of such technologies (ITRC 2019).

ASC was largely a precursor to Triad which was proposed nearly 20 years ago (Crumbling et al. 2003). This was an approach that sought to reduce delays and costs of site investigations by limiting the number of mobilizations to sites in determining initial site data and site conceptualization by creating a framework and approach that allowed more rapid on-site decision making. Strategies to accelerate site contamination assessment were cast in terms of three primary actions: (i) systematic project planning, (ii) dynamic work plan strategies, and (iii) on-site real-time measurement technologies. ITRC (2007) provides guidance on Triad implementation. Systematic planning sought to ensure that the level of detail in project planning matched the intended use of the data being collected, so it accorded and improved the site CSM but also to serve the goals of the investigation. A dynamic work strategy required flexibility, on-ground expertise in a number of technical areas and relied on real-time data to reach decision points. It established a decision-tree linked to real-time uncertainty management practices and allowed changed decisions and updates to the CSM as information became available on-site. On-site analytical tools, rapid sampling platforms, and on-site interpretation and management of data made dynamic work strategies possible. For investigations, apart from geoprobe and geophysical approaches, methods might have included hand-held field devices, and mobile transportable laboratories. Although not on-site nor in real time, recently Ciampi et al. (2022) sought to integrate surface and downhole geophysics with other site information to create a 3D image of the subsurface at a petroleum impacted site.

Despite efforts, implementation of Triad has been limited, with staged investigations and multiple mobilizations to sites still current. A paradigm shift is warranted. Without some knowledge of the subsurface to create an initial CSM, it appears difficult to know a priori the scale of effort required for a Triad approach. With an established CSM, we can do better at automating our decision making. This may predicate the need for an initial stage of investigation or modelling which establishes a CSM and the risk profile of a site (as per standard staged approaches or via Triad/ASC). Accelerating investigations toward automation may be more applicable to tracking remedial actions and perhaps longer-term management of such sites. This is where sensors, passive devices, geophysics and digital twins may play an increasingly important role.

## Possible way forward

**Fig. 2:**
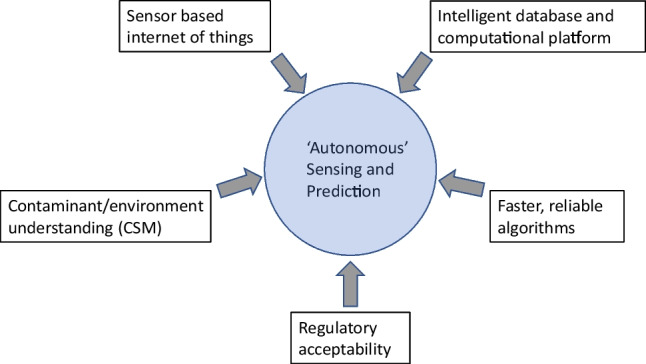
Depiction of important criteria for advancing autonomous sensing and prediction of subsurface pollution. Note that CSM is the conceptual site model

To be able to automate monitoring and critical decision making for management of contaminated soil and groundwater, apart from demonstration of measurement and modelling technology feasibility and viability, several criteria may need to be satisfied (Fig. 2):

1. Deep understanding of specific contaminant/environmental coupling embedded in a mature conceptual site model,
2. Measurement/sensor technologies specific to the contaminant and processes in the subsurface,
3. Intelligent data handling linked to a computational platform embodying relevant key subsurface processes and where necessary realizations of a site’s heterogeneous subsurface – reliant on computational and digital storage and handling speeds and capacity relevant to the application,
4. Fast and reliable algorithms to estimate and predict subsurface behaviours and chemical fate, without undue embedded uncertainty, and
5. Regulatory acknowledgement and comfort with likely outcomes

### Deep understanding of contaminant/environmental coupling

This is needed to prioritize the key properties of the contaminant and the environment that would allow targeted sensing and an understanding of controlling processes. Deep understanding and conceptualization come from identification of the key processes governing contaminants in the subsurface. Different chemicals and contaminants have different properties and will require different targeting—e.g., NAPLs might be light (LNAPLs) or dense (DNAPLs), and may be made up of thousands of compounds like petroleum or may be single component NAPLs like the solvent trichloroethene; VOCs may be resident in soil gas; highly solubility compounds might continue to feed groundwater plumes; organics may sorb or partition more to natural organic carbon in soils/aquifers; compounds may be pH dependent; compounds like PFAS may partition to air–water interfaces in soils; highly degradable compounds might consume or produce daughter products and/or gases and deplete electron donors/acceptors; and biota might adapt and respond to chemical contaminants (Murphy et al 2022). Some knowledge of these, and other site aspects, are needed to create a high-quality CSM. For some contaminants and systems, this may rely on considerable prior research and knowledge. For example, there has been decades of research on petroleum hydrocarbons and solvents in the subsurface (Davis et al. 2022), but significantly less on PFAS compounds and their additional air–water interfacial behaviours (Brusseau 2018). Ongoing research will create deeper understanding and improved CSMs to enable focused efforts and automated sensing. As such automating sensing and decisions for PFAS management may not yet be feasible.

### Measurement/sensor technologies

These have been described earlier. Whilst surrogates such as reduction potential changes have been deployed to investigated groundwater remedial processes (Blotevogel et al. 2021), missing or less well-developed technologies are contaminant specific sensors that are readily deployable into the subsurface, and robust enough to have a prolonged sensor life. Ideally, probes would consist of such sensors that auto-analyze chemical trends and telemetry data directly back from the subsurface to an enabled data base attached to an interpretive computational platform. VOC probes as described by Patterson et al (2000b) allow robust prolonged measurement but are not compound specific if a mixture of VOCs is present in the subsurface as with petroleum fuels. Achieving low concentration compliance criteria such as needed for benzene and some PFAS may also be beyond current devices. Efforts are needed to create specific sensors, which may involve linking material science to electrochemistry (Sakale et al. 2011) and/or novel biosensors (Komori et al. 2009; Cai et al. 2022) to create such probes. Additionally, pattern recognition machine learning promises intelligent sensor/detector applications where complex chemical signals may be measured, as explored by Ballard et al. (2021). Telemetering data directly back from subsurface sensors would seem improbable, but investigations are pursuing such an advancement especially related to agricultural practices (Huang et al. 2020; Levintal et al. 2022). As with any method, calibration of sensors and avoiding drift remains challenging if probes/sensors are to be deployed over long periods. Advancements in powering sensors are also needed. A field application of a current sensor network deployment and its usefulness and limitations is given in the case study that follows.

### Intelligent data handling linked to a computational platform

Telemetered sensor data needs to be captured, catalogued, and curated in an interpretive data handling system. Linkage to a computational platform that encompasses key processes and physicochemical and bio controls could also provide constraints on data interpretation. Such capability can lead to an auto-calibrated model of subsurface processes. Zhang and Cheng (2021) describe an intelligent collection vehicle system based on 5G communication technology that was applied to the data analysis of polluted sites. Simple interpretive tools would include spatial interpolation of data (e.g., subsurface groundwater, soil or gas plumes, temperature, soil moisture variations), calculation of simple metrics from available data (e.g., degradation rates, mass in the subsurface, temperature corrections), and display and interpretation of sensor trends (e.g., as a sentinel of pollution impacts, as a trigger to enhance or decrease active remedial efforts). Whilst interpretive, critical too is future proofing large-scale data storage and its long-term accessibility, a topic of discussion for many years (Findlay 2002).

Additionally, in key cases, the interpretation of data coming from rapid field measurements and sensors can be constrained and informed by linkage to a biogeochemically-based computational platform of key underlying processes such as proposed by Sookhak Lari et al. (2022). Such platforms embody key known features of the CSM, updated by such sensor/measurement inputs. To establish viable linked sensor-modelling platforms will require expanded computational and digital capacity. New supercomputers and coupling of multiple high-speed hardware nodes will continue to create such capacity. Quantum computing, when enabled, offers to accelerate run times by orders of magnitude and makes implementation of such sensor-modelling platform linkages viable for automated management and optimization problems (Gamble 2019). However, quantum computing remains some time away.

### Faster and reliable algorithms

Regardless of computational capacity advances, modelling linkages across all processes and three-dimensional subsurface heterogeneity is computationally intensive, leading to prolonged model run times. Faster, yet still reliable algorithms that approximate subsurface processes with less computational overhead are needed. Modelling that accommodates most of the site and chemical interaction complexities allows assessment of model output sensitivities; providing insights that allow simplifying assumptions about the relative importance of processes and parameters. This in turn can allow the removal of computationally burdensome but less important processes that do not significantly influence model estimations. In one example, for estimation of LNAPL thicknesses in the subsurface, Lenhard et al. (2018) compared a simplified multiphase estimation approach to that of a complex multiphase code, finding good correspondence and in so doing establishing a practical and reliable tool for management decisions without the overheads of numerical multiphase modelling. Another example is that of Davis et al. (2009b), whereby, based on field data, biodegradation of petroleum vapours in vadose zone soils was observed to occur rapidly compared to transport processes, and was approximated as an instantaneous rate, leading to the assumption of all vapour and oxygen consumption being at a single point in the soil profile. This reduced the number of parameters and processes considerably, allowing the effects of buildings to be represented simply (Davis et al. 2021), with outcomes independent of soil type, moisture content or reaction kinetics. This is despite a single component vapour being assumed and layering not being accommodated in the model. These examples show an otherwise complex system to be represented somewhat simply assisting in identifying which parameters are less important to continue to include in a model simulation. However, this is not to deny uncertainty and that subsurface systems can have greater levels of complexity through heterogeneity and temporal influences.

### Regulatory acceptance

Automating field monitoring and reporting via sensors and linked models may challenge regulatory acceptance. Standardized and staged investigations are the accepted approach (ASTM 1998; NEPM 2013; HEPA 2020). While novel approaches are somewhat encouraged, regulatory acceptance would need appropriate validation with the level of uncertainty quantified. For chemicals with low environmental acceptance criteria (such as PFAS; HEPA 2020) sensors might need to be capable of detecting such low concentrations or sensors could target alternate indicators that would be surrogates for low concentrations. Again, currently standardized sampling of groundwater, soil or soil gas would be expected with analysis undertaken in qualified laboratories, with appropriate duplicate/triplicate, trip blanks and other quality assurance and quality control measures on the analysis. Deep understanding (as articulated earlier) and appropriate field validation and peer review of technology advances may lead to greater acceptance and uptake in a regulatory compliance framework. To achieve this, prosecution of a global program of technology advancement and validation is likely needed to integrate sensors into critical compliance decisions.

## NSZD monitoring and prediction as an example case study

We consider LNAPL NSZD at a field site where sensors are deployed as an example case study of what is current and what might be achievable to create an autonomous sensing and prediction platform. We provide an overview of the field site and step though the five criteria (Fig. 2) to illustrate a possible pathway forward.

### Deep understanding and a mature CSM

We choose LNAPL NSZD since the processes of partitioning of LNAPL components to groundwater and soil gas phases and their biodegradation are becoming well understood as mass loss mechanisms, as per the reviews of Garg et al. (2017) and Sookhak Lari et al. (2019). Standard parameters that are measured to estimate NSZD rates are subsurface soil gas composition (oxygen, carbon dioxide, methane, VOCs), temperature and surface fluxes of carbon dioxide (ITRC 2009; CRC CARE 2018)—all being indicators of or consumed in biodegradation processes. As such the conceptualization of LNAPL NSZD processes are largely in place after decades of research—so addresses Criteria 1, which is the need for deep understanding. Additionally, the field site is the BP Kwinana site as described in Davis et al. (2022), which has a mature CSM, with investigations spanning decades (e.g., Davis et al. 1998). Petroleum hydrocarbon masses across the site have been reasonably quantified and the depth distribution of LNAPL within the shallow sandy soil profile and in the zone of water table fluctuation 2–5 m below ground surface.

### Measurement technologies

To use accepted methods to estimate NSZD rates, VOC, oxygen, and carbon dioxide probes (Patterson et al. 2000b; Patterson and Davis 2008; Patterson et al. 2013) as well as thermistors and soil moisture probes were installed at multiple depths and locations at the Kwinana site. Duplicate strings of gas probes and thermistors were installed ~ 20 m apart at each location specific to crude oil, gasoline, diesel, aviation gasoline and a background location. Manual multi-depth samplers were also installed to verify probe data, and a third set of multi-depth samplers were installed between the probe locations (all within 20 m distance of each other). The field sensors are actively controlled by infield multichannel data loggers (Datataker) that can be adjusted remotely to alter sampling frequency, as well as used to log gas flow rates in the lines of those that require carrier gas flows (the VOC and CO_2_ probes), with a reduction in flow being an indicator of loss of pressure in the lines. Advances in sensors would ideally do away with circulating gases and other infrastructure complexities.

**Fig. 3:**
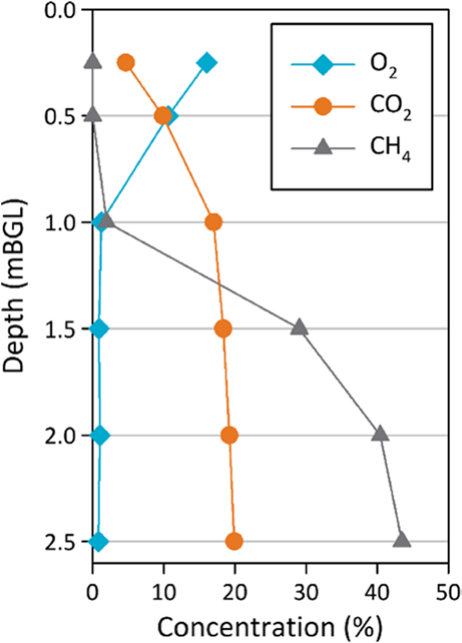
Soil gas depth measurements (manual) of oxygen (O_2_), carbon dioxide (CO_2_), and methane (CH_4_) at the aviation gasoline site on 26 October 2020, for the multilevel sampler location VZ03

Indicative manually sampled data for the aviation gasoline location is shown in Fig. 3. Aviation gasoline LNAPL was distributed from 3.0 to 4.0 m below ground level (bgl). The manual measurements showed the oxygen concentration decreased to zero at 1 m bgl from near atmospheric concentrations (21%) at 0.25 m bgl. Carbon dioxide decreased from about 20% at a 2.5 m depth to low levels at shallow soil depths. The methane concentration was about 43% at a 2.5 m depth and decreased towards the surface to effectively zero at 1 m bgl coincident with zero oxygen concentrations. This aligns with our conceptualization of the subsurface NSZD processes whereby oxygen will be consumed during the biodegradation of methane in the soil profile and in doing so generate carbon dioxide.

**Fig. 4:**
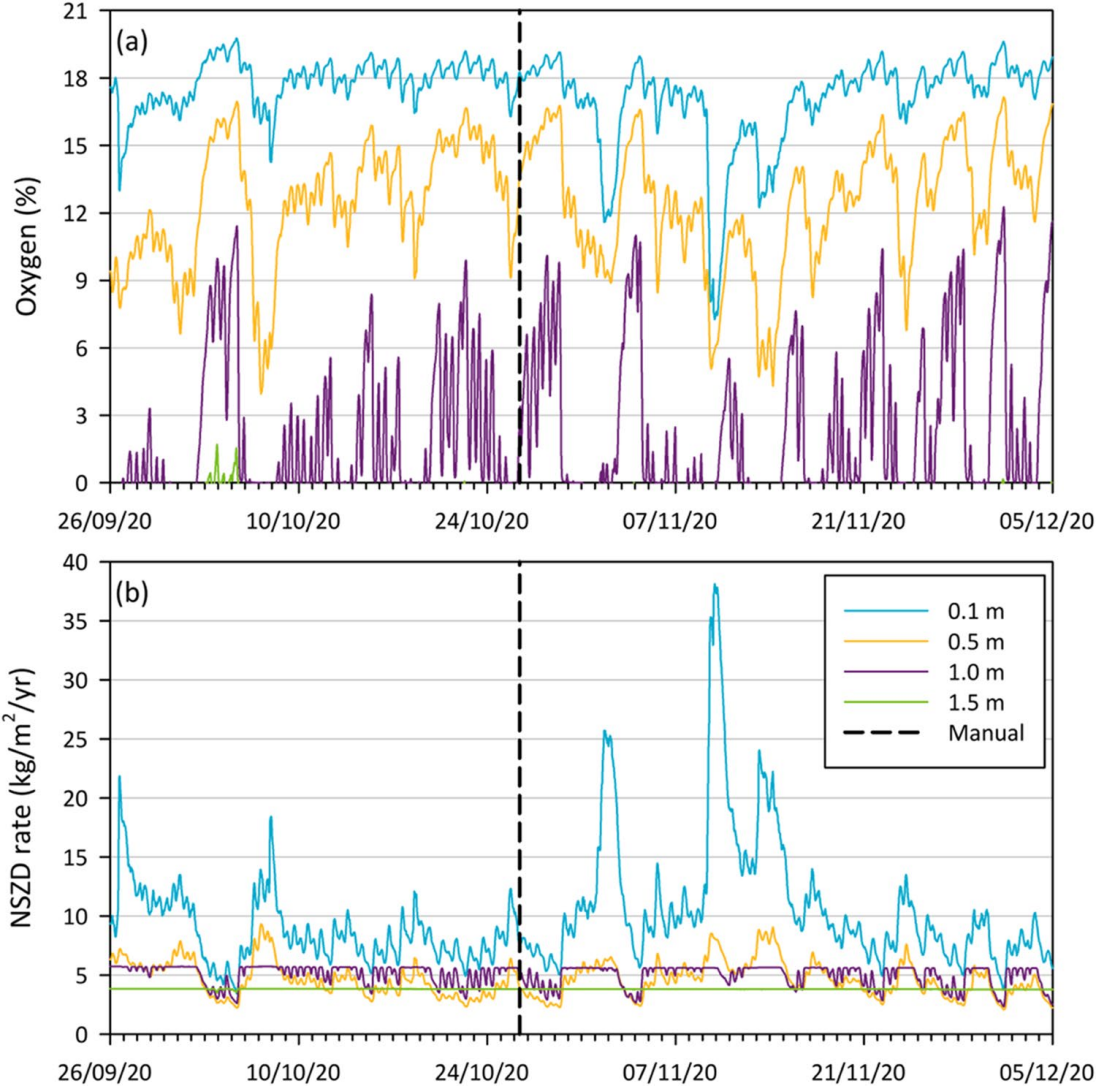
September to December 2020 time series of (a) oxygen concentrations from oxygen probes buried at 0.1, 0.5, 1.0, 1.5 m depths below ground, and (b) NSZD rates determined from the oxygen probe data from each of these depths. The vertical dashed line is the date of sampling for the data depicted in Fig. 3

Over an approximate 2-month period in September–December 2020, Fig. 4a shows oxygen concentration time series for O_2_ probes adjacent to VZ03 at 0.1, 0.5, 1.0, and 1.5 m depths below ground. The date of manual sampling of VZ03 in Fig. 3, is depicted in Fig. 4 by a vertical dashed line. This shows that the O_2_ probe data correlates well to the depth distribution determined by manual sampling (Fig. 3), providing verification of the data. For the probe data (Fig. 4a), oxygen concentrations are seen to fluctuate less at shallower depths (e.g., at 0.1 m) than at deeper depths. This might be expect given the fixed atmospheric concentration in air at ground surface, although sharp oxygen concentration decreases at 0.1 m were observed around the 1^st^ and again on the 10^th^ November.

Concentration variations change the vertical O_2_ gradient, which is used to calculate NSZD rate estimates. For the 15-min interval times series, Fig. 4b shows NSZD rate estimates calculated from the O_2_ concentration data set in Fig. 4a for data from each probe depth, and assuming a constant diffusion coefficient over this time period (2.3 × 10^–6^ m^2^/s for oxygen, being the minimum proposed by Davis et al. 2022). In reality, the diffusion coefficient will vary based on variations in soil moisture content, but such refinements are not included here. We are only seeking to illustrate the point that it is possible from such sensors to provide automated high-frequency data and calculations for a key management measure if probe data are ported to an intelligent database with such embedded capability. With these assumptions the NSZD rates can be determined on a 15-min basis and reported automatically—showing significant increases and decreases across a threefold change using deeper oxygen probe data (2.5–7.5 kg/m^2^/y – equivalent to ~ 40,000–120,000 L/ha/y) and perhaps tenfold if the 0.1 m depth probe data are used.

Again, what is shown is for illustration purposes. Not shown are online CO_2_, VOC, nor temperature data, which are all linked to the consumption of O_2_ and as such would create additional estimates of NSZD rates and, upon intercomparison, lead to constraints on the ranges of NSZD rates generated from such online probe data. As indicated, being able to do this creates greater confidence and certainty on estimates that might be used for management and greater confidence for regulatory purposes. For example, based on the rates in Fig. 4b, and taking the specific mass at the site to be in the range of 15–68 kg/m^2^ (Davis et al. 2022), Fig. 4b could instead depict the estimated time to degrade all the aviation gasoline NAPL resident in the subsurface (the ranges here would be < 1–35 years with an approximate average of say 5–15 years)—a key measure for regulators and industry. Importantly with such sensors, changes can be logged over years to determine if rates decrease (e.g., Davis et al. 2022) and NAPL longevity is prolonged. Note that if the maximum oxygen diffusion coefficient proposed in Davis et al. (2022) were used here rates would be approximately double those in Fig. 4b, and NAPL longevity would be halved.

Challenges and shortcomings include:

- Lateral spatial variability of the subsurface is likely due to LNAPL variability or soil textural changes over short intervals. This could be captured by deployment of additional sets of in situ sensors at appropriate lateral distances. Spatial textural or soil/aquifer structural changes might be determined using in ground, downhole or surface geophysical methods (Barber et al. 1991; García-Rincón et al. 2020) or perhaps drone or satellite mounted sensors (Roy et al. 2017; Kelleher et al. 2018).
- As currently configured the VOC probes measure the full mixture of volatiles and so for this petroleum example would not individually report a critical risk driver such as benzene, but that may not be a critical issue if the VOC probes were deployed for single VOC contaminants such as trichloroethene (Benker et al. 1997; Basu et al. 2009) of if benzene was shown by other measures (such as oxygen) to be degrading adequately (Davis et al. 2021).
- Similarly, the probes as configured did do not measure methane separately to VOCs. Where methane is thought to be a critical parameter and indicator of NSZD bioprocesses (Garg et al. 2017) this would be critical to advance.
- Soil moisture probes were installed but data were not overly reliable. These is critical to enable estimation of air-filled porosity and effective diffusion coefficient changes in the soil for automated reporting of long term NSZD rate estimates.

### Interpretive data handling system, linked to a computational platform

Currently this is not in place, although in part Fig. 4b indicates aspects of value adding and interpretation of the data that have been embedded in the data handling system. The data from the in-field sensors/loggers is telemetered via modems to a centralized, structured PostgreSQL database (Horsburgh et al. 2016). Database access, visualization of data and calculation of NSZD rates is undertaken manually, though some tasks have been semi-automated using scripts written in Python (van Rossum 2003). Creating software able to undertake such work automatically would not be overly ambitious and is currently feasible. Calculating NSZD rates from soil gas depth profiles is simply done as indicated via calculating the vertical oxygen, carbon dioxide, VOC and/or methane gradient in the soil vadose zone, multiplying by the effective diffusion coefficient (adjusted by the moisture content changes), and multiplied by the stoichiometry of the chemical equation for biodegradation of petroleum hydrocarbon (CRC CARE 2018). Rates could also be calculated from depth profiles of temperature data using thermal properties of the soil and the heat generated by the biodegradation processes. Linkage to an intelligent computational platform is also currently not in place, but a digital twin of current key NSZD processes is being progressed (Sookhak Lari et al. 2022). Establishment of an intelligent database and a digital twin of the Kwinana site is feasible. Further advances are needed to link them and self-calibrate the computational representation and rate estimations based on temporal sensor data.

### Fast and reliable algorithms

**Fig. 5:**
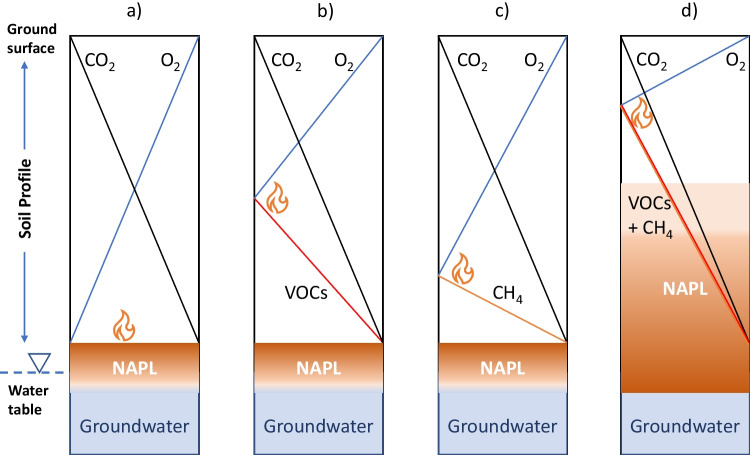
Depiction of possible gas depth profiles above (and within) a petroleum NAPL at steady-state where no layering or soil moisture variations are assumed, and no background natural soil carbon respiration processes are occurring. Note that the maximum scale for VOCs and methane would be different to oxygen and carbon dioxide. All show linear oxygen concentration decreases with depth and carbon dioxide increases with depth in the soil vadose zone due to NAPL biodegradation. The symbol  denotes the approximate depth where heat would be produced due to aerobic biodegradation processes. The panels depict a) where NAPL has no VOCs and methane is not produced; b) where NAPL has VOC vapours moving into the vadose zone but methane is not produced c) where NAPL has no VOCs but methane is being produced and d) where NAPL is at a shallower depth of the vadose zone and VOCs and methane may be present in the vadose zone

The NSZD digital twin prototype of Sookhak Lari et al. (2022) is complex and can be slow especially if expanded to 3D and used for optimization of management options. However, while still seeking to use such models of processes to help constrain possible interpretations from available sensor data, here we seek to generate value from the sensor array for ongoing estimation of NSZD rates. As indicated earlier, for some gas transport processes simplification is possible if say biodegradation is fast compared to transport processes (e.g., Davis et al. 2009b; Knight and Davis 2013). If these simplifications were combined with other measured data such as soil moisture and temperature, it may be possible to reduce a complex multiphase problem to one that simply couples gas transport and its consumption/production with the requisite heat production, adjusted by soil moisture changes. Possible conceptualizations leading to simplified models and algorithms are shown in Fig. 5. The conceptualization constitutes simply coupled counter-current gas transport, with heat conduction from associated biodegradation, avoiding complex nonlinear multiphase transport, partitioning and microbial growth and decay algorithms. To illustrate, if we take the data in Fig. 4, and apply the linear model in Fig. 5b, c then we estimate the NSZD rate relatively easily over time to give Fig. 4b reporting NSZD rates over this transient period in real time.

### Regulatory acceptance

NSZD is increasingly being established in management guidance (ITRC 2009; CL:AIRE 2019; CRC CARE 2020), with promulgated case studies and understanding (Rayner et al. 2020; Smith et al. 2022). Expressed uncertainties relate to undue risks (e.g., from benzene) and the longevity of the LNAPL in the subsurface posing a prolonged risk. As indicated earlier, developed sensors specific to benzene if deployed at an appropriate spatial coverage and logging at an acceptable frequency could alleviate such concerns. We note though that adoption of NSZD as a management option requires that all relevant risks are controlled or eliminated (CRC CARE 2020). Assessment of the longevity of LNAPL in the subsurface is a direct outcome of the deployment of sensors that allow mass losses to be logged frequently and over prolonged times. Regulatory acceptance is increasingly positive, and the LNAPL NSZD option is being accepted for LNAPL site management. As with any “standard” guidance unusual circumstances or overly complex geologies and heterogeneities might demand additional investigations or close management. Sensors can provide the online information if targeting the right parameters to maintain confidence in management. If variations are minimal over time, investments in and reliance on monitoring and sensor networks decrease.

## Conclusions

Investigation, measurement, and prediction technologies for pollutants in subsurface environments have advanced considerably over the last 40–50 years. National programs and efforts have been spurred by major pollution events (e.g., Love Canal over 40 years ago in 1978–1980), and again more recently with the occurrence of PFAS impacts and risks.

Global digitization and automation are accelerating. Subsurface monitoring and prediction to define risks and manage pollution instances is falling behind and requires investment. Sustainability imposts demand that we create more efficient management and remedial options, possibly offered by better sensing and automated data interrogation. Natural heterogeneity of soil and groundwater environments mitigates against advancing autonomous sensing and prediction of the environment.

However, advances in sensors specific to the contaminant/environmental context linked to digital twins simplified to the core processes, promise the potential to automate assessments and predictive capability—as sentinel capacity or to decide on new or revised actions to take at impacted sites. Five criteria for success have been articulated. A linked system may trigger and “decide” on the need for increased effort, management or remedial action, or it may signal, trigger or decide on reduced effort—for example where NSZD rates at a petroleum impacted site far exceed mass removal from active remediation, and where risks are contained. Critical to investment in deploying linked and automated systems is regulatory acceptance. The case study on LNAPL NSZD at a large field site shows that such an ambition may be feasible, albeit the case study does not have all aspects of all five criteria yet in place. Advancing this research is planned. Global investment in a program of technology advancement and validation is needed to integrate sensors into critical compliance decisions for subsurface pollution management.

## Data Availability

No data, material, or code is available associated with the manuscript.

## References

[CR1] Anderson RH (2021). The case for direct measures of soil-to-groundwater contaminant mass discharge at AFFF-impacted sites. Environ Sci Technol.

[CR2] Annable MD, Hatfield K, Cho J, Klammler H, Parker BL, Cherry JA, Rao PSC (2005). Field-scale evaluation of the passive flux meter for simultaneous measurement of groundwater and contaminant fluxes. Environ Sci Technol.

[CR3] ASTM (1998) Standard guide for accelerated site characterization for confirmed or suspected petroleum releases. Public No. E1912, Am Soc Testing Mater. www.astm.org. Accessed June 2022

[CR4] ASTM (2018) Standard guide for pore-liquid sampling from the Vadose Zone. ASTM D4696–18, ASTM International, West Conshohocken, PA, 2018. www.astm.org. Accessed June 2022

[CR5] Atekwana E, Atekwana E (2009). Geophysical signatures of microbial activity at hydrocarbon contaminated sites: A review. Surv Geophys.

[CR6] Ballard Z, Brown C, Madni AM, Ozcan A (2021). Machine learning and computation-enabled intelligent sensor design. Nature Mach Intell.

[CR7] Barber C, Briegel D (1987). A method for the in-situ determination of dissolved methane in groundwater in shallow aquifers. J Contam Hydrol.

[CR8] Barber C, Davis GB (1987). Representative sampling of groundwater from short-screened boreholes. Ground Water.

[CR9] Barber C, Davis GB, Briegel D, Ward JK (1990). Factors controlling the concentration of methane and other volatiles in groundwater and soil-gas around a waste site. J Contam Hydrol.

[CR10] Barber C, Davis GB, Farrington P (1990b) Sources and sinks for dissolved oxygen in groundwater in an unconfined sand aquifer, Western Australia. In: Durrance EM et al (eds) Geochemistry of gaseous elements and compounds. Theophrastus Public, SA, pp 353–368

[CR11] Barber C, Davis GB, Buselli G, Height M (1991). Remote monitoring of groundwater pollution using geo–electric techniques in undulating sandy terrain, Western Australia. Int J Environ Pollut.

[CR12] Basu NB, Rao PSC, Poyer IC, Nandy S, Mallavarapu M, Naidu R, Davis GB, Patterson BM, Annable MD, Hatfield K (2009). Integration of traditional and innovative characterization techniques for flux-based assessment of dense non-aqueous phase liquid (DNAPL) sites. J Contam Hydrol.

[CR13] Benker E, Davis GB, Barry DA (1997). Factors controlling the distribution and transport of trichloroethene in a sandy aquifer - hydrogeology and results of an in situ transport experiment. J Hydrology.

[CR14] Berho C, Togola A, Coureau C, Ghestem JP, Amalric L (2013). Applicability of polar organic compound integrative samplers for monitoring pesticides in groundwater. Environ Sci Pollut Res Int.

[CR15] Binley A, Cassiani G, Deiana R (2010). Hydrogeophysics: opportunities and challenges. Bollettino Di Geofisica Teorica Ed Applicata.

[CR16] Blotevogel J, Askarani K, Hanson A, Gallo S, Carling B, Mowder C, Spain J, Hartten A, Sale T (2021). Real-time remediation performance monitoring with ORP sensors. Groundw Monit Remediat.

[CR17] Brusseau ML (2018). Assessing the potential contributions of additional retention processes to PFAS retardation in the subsurface. Sci Total Environ.

[CR18] Brusseau ML, Anderson RH, Guo B (2020). PFAS concentrations in soils: background levels versus contaminated sites. Sci Total Environ.

[CR19] Burton JC, Walker JL, Jennings TV, Aggarwal PK, Hastings B, Meyer WT, Rose CM, Rosignolo CL (1993) Expedited site characterization: a rapid, cost-effective process for preremedial site characterization. Proceedings of United States Superfund 14 conference, Washington DC. https://www.osti.gov/servlets/purl/10104559. Accessed Jan 2023

[CR20] Buselli G, Davis GB, Barber C, Height MI, Howard SHD (1992). The application of electromagnetic and electrical methods to groundwater problems in urban environments. Explor Geophys.

[CR21] Buselli G, Barber C, Davis GB, Salama RB (1990) Detection of groundwater contamination near waste disposal sites with transient electromagnetic and electrical methods. In: Ward SH (ed) Geotechnical and environmental geophysics vol II: Environmental and groundwater. SEG Public, pp 27–39. 10.1190/1.9781560802785.2

[CR22] Cai Y, Zhu K, Shen L, Ma J, Bao L, Chen D, Wei L, Wei N, Liu B, Wu Y, Chen S (2022). Evolved biosensor with high sensitivity and specificity for measuring cadmium in actual environmental samples. Environ Sci Technol.

[CR23] Christy TM (1996) A driveable permeable membrane sensor of volatile compounds in soil. Tenth National Outdoor Action Conference, Dublin, Ohio. 10.3997/2214-4609-PDB.203.1998_007

[CR24] Ciampi P, Esposito C, Cassiani G, Deidda GP, Flores-Orozco A, Rizzetto P, Chiappa A, Bernabei M, Gardon A, Papini MP (2022). Contamination presence and dynamics at a polluted site: Spatial analysis of integrated data and joint conceptual modeling approach. J Contam Hydrol.

[CR25] CityChlor (2013) Groundwater quality measurement with passive samplers – Code of best practices. INERIS reference: DRC-13–102468–03494A, 66 pp. CityChlor - Rijkswaterstaat Environment (https://rwsenvironment.eu) Accessed June 2022

[CR26] CL:AIRE (2019) An introduction to Natural Source Zone Depletion at LNAPL sites. CL:AIRE Technical Bulletin TB20. CL:AIRE, London. https://www.claire.co.uk/home/news/1176-nszd-bulletin. Accessed May 2022

[CR27] Clement TP, Gautam TR, Lee KK, Truex MJ, Davis GB (2004). Modeling of DNAPL-dissolution, rate-limited sorption and biodegradation reactions in groundwater systems. Bioremediat J.

[CR28] CLU-IN (2022) Contaminated Site Clean-Up Information (CLU-IN). US Government and EPA linked website: https://clu-in.org. Accessed June 2022

[CR29] CRC CARE (2018) Technical measurement guidance for LNAPL natural source zone depletion. CRC CARE Technical Report no. 44, CRC for Contamination Assessment and Remediation of the Environment, Newcastle, Australia. https://crccare.com/technical-reports/

[CR30] CRC CARE (2020) The role of natural source zone depletion in the management of light non-aqueous phase liquid (LNAPL) contaminated sites. CRC CARE Technical Report no. 46, CRC for Contamination Assessment and Remediation of the Environment, Newcastle, Australia. https://crccare.com/technical-reports/

[CR31] Crumbling DM, Griffith J, Powell DM (2003). Improving decision quality: Making the case for adopting next generation site characterization practices. Remediation.

[CR32] Dahan O (2020) Vadose zone monitoring as a key to groundwater protection. Front Water 2. 10.3389/frwa.2020.599569

[CR33] Davis GB, Johnston CD, Thierrin J, Power TR, Patterson BM (1993). Characterising the distribution of dissolved and residual NAPL petroleum hydrocarbons in unconfined aquifers to effect remediation. J Aust Geol Geophys.

[CR34] Davis GB, Johnston CD, Patterson BM, Barber C, Bennett M (1998). Estimation of biodegradation rates using respiration tests during in situ bioremediation of weathered diesel NAPL. Ground Water Monit Rem.

[CR35] Davis GB, Patterson BM, Johnston CD (2009). Aerobic bioremediation of 1,2 dichloroethane and vinyl chloride at field scale. J Contam Hydrol.

[CR36] Davis GB, Patterson BM, Trefry MG (2009). Evidence for instantaneous oxygen-limited biodegradation of petroleum hydrocarbon vapours in the subsurface. Ground Water Monit Rem.

[CR37] Davis GB, Knight JH, Rayner JL (2021). Extinguishing petroleum vapor intrusion and methane risks for slab-on-ground buildings: a simple guide. Groundw Monit Remediat.

[CR38] Davis GB, Rayner JL, Donn MJ, Johnston CD, Lukatelich R, King A, Bastow TP, Bekele E (2022). Tracking NSZD mass removal rates over decades: Site-wide and local scale assessment of mass removal at a legacy petroleum site. J Contam Hydrol.

[CR39] Davis GB, Rayner JL (2021) Advancing direct vadose zone measures of soil-to-groundwater PFAS pore water concentrations using lysimeters. CSIRO Land Water Tech Rep, November 2021 (available from authors)

[CR40] Davis GB, Barber C, Buselli G, Sheehy A (1991) Potential applications to monitoring remediation in Australia using geoelectric and soil-gas techniques. In: Hinche RE, Olfenbuttel RF (eds) In-Situ Bioreclamation: Applications and Investigations for Hydrocarbon and Contaminated Site Remediation, Butterworth-Heinemann, Boston, pp 337–350

[CR41] Davis GB, Merrick NP, McLaughlan RG (2006) Protocols and techniques for characterising sites with subsurface petroleum hydrocarbons – a review. CRC CARE Technical Report no. 2, CRC for Contamination Assessment and Remediation of the Environment, Adelaide, Australia, 79 pp. https://crccare.com/technical-reports/

[CR42] Diran MO, Phillips JA (1994) Interactive soil gas survey technique for vadose zone plume delineation. United States. Proceedings Federal Environmental Restoration and Waste Minimization conference and exhibition: 2:858. https://www.osti.gov/biblio/124579

[CR43] Engelmann C, Sookhak Lari K, Schmidt L, Werth CJ, Walther M (2021). Towards predicting DNAPL source zone formation to improve plume assessment: Using robust laboratory and numerical experiments to evaluate the relevance of retention curve characteristics. J Haz Mater.

[CR44] Findlay C (2002). Future proof: ensuring the long-term accessibility of technology-dependent records. Rec Manag J.

[CR45] Gamble S (2019) Quantum computing: What it is, why we want it, and how we’re trying to get it. In: National Academy of Engineering. Frontiers of Engineering: Reports on Leading-Edge Engineering from the 2018 Symposium. Washington (DC): National Academies Press (US). https://www.ncbi.nlm.nih.gov/books/NBK538701/. Accessed Mar 2022 30883073

[CR46] García-Rincón J, Gatsios E, Rayner JL, McLaughlan RG, Davis GB (2020). Laser-Induced Fluorescence logging as a high-resolution characterisation tool to assess LNAPL mobility. Sci Tot Environ.

[CR47] Garg S, Newell CJ, Kulkarni PR, King DC, Adamson DT, Renno I, Sale T (2017). Overview of natural source zone depletion: processes, controlling factors, and composition change. Groundw Monit Remediat.

[CR48] Gelernter DH (1991) Mirror worlds: or the day software puts the universe in a shoebox — How it will happen and what it will mean. Oxford University Press, Oxford

[CR49] Gliński J, Stępniewski W (1985) Soil aeration and its role for plants, 1st edn. CRC Press, Boca Raton

[CR50] Grieves M (2019) Virtually intelligent product systems: digital and physical twins. In: Flumerfelt S et al (eds) Complex systems Eengineering: Theory and practice American Institute of Aeronautics and Astronautics, pp 175–200

[CR51] Gunn A (2020) Living in a digital world: the causes and the consequences. Digital Society, Web page (Living in a digital world: the causes and the consequences | by Amelia Gunn | Digital Society | Medium) Accessed June 2022

[CR52] Ha J-H, Seagren EA, Song X (2014) Oxygen transport across the capillary fringe in LNAPL pool-source zones. J Environ Engineering 140(12). 10.1061/(ASCE)EE.1943-7870.0000866

[CR53] Hale SE, Canivet B, Rundberget T, Langberg HA, Allan IJ (2021) Using passive samplers to track Per and Polyfluoroalkyl Substances (PFAS) emissions from the paper industry: laboratory calibration and field verification. Frontiers in Environmental Science https://www.frontiersin.org/article/10.3389/fenvs.2021.796026

[CR54] Hartmann H, Hefner C, Carter E, Liles D, Divine C, Edmiston PL (2021). Passive sampler designed for per- and polyfluoroalkyl substances using polymer-modified organosilica adsorbent. AWWA Water Sci.

[CR55] HEPA (2020) PFAS National Environmental Management Plan, version 2.0. National Chemicals Working Group of the Heads of EPAs Australia and New Zealand January 2020. https://www.dcceew.gov.au/environment/protection/publications/pfas-nemp-2. Accessed June 2022

[CR56] Horsburgh JS, Aufdenkampe AK, Mayorga E, Lehnert KA, Hsu L, Song L, Jones AS, Damiano SG, Tarboton DG, Valentine D, Zaslavsky I, Whitenack T (2016). Observations data model 2: a community information model for spatially discrete Earth observations. Environ Model Software.

[CR57] Huang H, Shi J, Wang F, Zhang D, Zhang D (2020). Theoretical and experimental studies on the signal propagation in soil for wireless underground sensor networks. Sensors.

[CR58] Hutson JL, Wagenet RJ (1995) An overview of LEACHM: A process-based model of water and solute movement, transformations, plant uptake and chemical reactions in the unsaturated zone. In: Loeppert RH et al (eds) Chemical equilibrium and reaction models, soil science society of America. 10.2136/sssaspecpub42.c19

[CR59] Hwang YK, Endres AL, Piggott SD, Parker BL (2008). Long-term ground penetrating radar monitoring of a small volume DNAPL release in a natural groundwater flow field. J Contam Hydrol.

[CR60] ITRC (2007) Triad Implementation Guide. SCM-3. Washington, D.C.: Interstate Technology & Regulatory Council; Sampling, Characterization, and Monitoring Team, 63 pp. www.itrcweb.org. Accessed May 2022

[CR61] ITRC (2009) Evaluating Natural Source Zone Depletion at sites with LNAPL. Interstate Technology Regulatory Council, Washington, D.C. Accessed at: https://itrcweb.org/viewdocument/evaluating-natural-source-zone-depl. Accessed Apr 2022

[CR62] ITRC (2019) Implementing advanced site characterization tools. The Interstate Technology and Regulatory Council (ITRC), p 328. www.itrcweb.org. Accessed Mar 2022

[CR63] Jawitz JW, Annable MD, Clark CJ, Puranik S (2002). Inline gas chromatographic tracer analysis: An alternative to conventional sampling and laboratory analysis for partitioning tracer tests. Instrum Sci Technol.

[CR64] Johnston CD, Trefry MG (2009). Characteristics of light nonaqueous phase liquid recovery in the presence of fine-scale soil layering. Water Resour Res.

[CR65] Johnston CD, Rayner JL, Patterson BM, Davis GB (1998). The contribution of volatilisation and biodegradation during air sparging of dissolved BTEX-contaminated groundwater. J Contam Hydrol.

[CR66] Kelleher C, Scholz CA, Condon, L, Reardon, M (2018) Drones in geoscience research: the sky is the only limit. Eos 99. 10.1029/2018EO092269

[CR67] Knight JH, Davis GB (2013). A conservative vapour intrusion screening model of oxygen-limited hydrocarbon vapour biodegradation accounting for building footprint size. J Contam Hydrol.

[CR68] Komori K, Miyajima S, Tsuru T, Fujii T, Mohri S, Ono Y, Sakai Y (2009). A rapid and simple evaluation system for gas toxicity using luminous bacteria entrapped by a polyion complex membrane. Chemosphere.

[CR69] Laor Y, Ronen D, Graber ER (2003). Using a passive multilayer sampler for measuring detailed profiles of gas-phase VOCs in the unsaturated zone. Environ Sci Technol.

[CR70] Lenhard RJ, Sookhak Lari K, Rayner JL, Davis GB (2018). Evaluating an analytical model to predict subsurface LNAPL distributions and transmissivity from current and historic fluid levels in groundwater wells: Comparing results to numerical simulations. Groundwat Monit Remediat.

[CR71] Levintal E, Ganot Y, Taylor G, Freer-Smith P, Suvocarev K, Dahlke HE (2022). An underground, wireless, open-source, low-cost system for monitoring oxygen, temperature, and soil moisture. SOIL.

[CR72] Li P, Karunanidhi D, Subramani T, Srinivasamoorthy K (2021). Sources and consequences of groundwater contamination. Arch Environ Contam Toxicol.

[CR73] Liang W, Abidi M, Carrasco L, McNelis J, Tran L, Li Y, Grant J (2020). Mapping vegetation at species level with high-resolution multispectral and lidar data over a large spatial area: A case study with Kudzu. Remote Sensing.

[CR74] Marrin DL, Kerfoot HB (1988). Soil-gas surveying techniques. Environ Sci Technol.

[CR75] Martin H, Patterson BM, Davis GB, Grathwohl P (2003). Field trial of contaminant groundwater monitoring. Comparing time-integrating ceramic dosimeters and conventional water sampling. Environ Sci Technol.

[CR76] Mehmood MZ, Ahmed M, Afzal O, Aslam MA, Zoq-ul-Arfeen R, Qadir G, Komal S, Shadid MA, Awan AA, Awale MA, Sameen A, Kalsoom T, Nasim W, Fayyaz-ul-Hassan, Ahmad S (2022) Internet of Things (IoT) and Sensors Technologies in Smart Agriculture: Applications, Opportunities, and Current Trends. In: Jatoi WN, Mubeen M, Ahmad A, Cheema MA, Lin Z, Hashmi MZ (eds) Building Climate Resilience in Agriculture. Springer, Cham. 339–364. 10.1007/978-3-030-79408-8_21

[CR77] Menger RF, Funk E, Henry CS, Borch T (2021). Sensors for detecting per- and polyfluoroalkyl substances (PFAS): A critical review of development challenges, current sensors, and commercialization obstacles. Chem Engineering J.

[CR78] Murphy CWM, Davis GB, Rayner JL, Walsh T, Bastow TP, Butler AP, Puzon GJ, Morgan MJ (2022). The role of predicted chemotactic and hydrocarbon degrading taxa in natural source zone depletion at a legacy petroleum hydrocarbon site. J Haz Mater.

[CR79] Namiesnik J, Zabiegala B, Kot-Wasik A, Partyka M, Wasik A (2005). Passive sampling and/or extraction techniques in environmental analysis: a review. Anal Bioanal Chem.

[CR80] NEPM (2013) National Environment Protection (Assessment of Site Contamination) Amendment Measure 2013. Schedule B2, “Guideline on Site Characterisation”, National Environment Protection Council, Australia. https://www.legislation.gov.au/Details/F2013C00288. Accessed May 2022

[CR81] Patterson BM, Davis GB (2008). An in situ device to measure oxygen in the vadose zone and in ground water: laboratory testing and field evaluation. Ground Water Monit Rem.

[CR82] Patterson BM, Power TR, Barber C (1993). Comparison of two integrated methods for the collection and analysis of volatile organic compounds in ground water. Groundw Monit Remediat.

[CR83] Patterson BM, Franzmann PD, Rayner JL, Davis GB (2000). Combining coring and suction cup data to improve the monitoring of pesticides in sandy vadose zones: a field-release experiment. J Contam Hydrol.

[CR84] Patterson BM, Davis GB, McKinley AJ (2000b) Volatile organic compounds in groundwater, probes for the analysis of. In: Meyers RA (ed) Encyclopedia of analytical chemistry: Instrumentation and application. John Wiley and Sons Ltd, New York, pp 3515–3526. 10.1002/9780470027318.a0882

[CR85] Patterson BM, Robertson BS, Woodbury RJ, Talbot B, Davis GB (2006). Long-term evaluation of a composite cover overlaying a sulfidic tailings facility. Mine Water Environ.

[CR86] Patterson BM, Furness A, Bastow TP (2013). Soil gas carbon dioxide probe: Laboratory testing and field evaluation. Environ Sci Process Impacts.

[CR87] Phillips AS, Hung Y-T, Bosela PA (2007) Love Canal tragedy. J Perform Constr Facilit 21(4). 10.1061/(ASCE)0887-3828(2007)21:4(313)

[CR88] Pinasseau L, Wiest L, Volatier L, Fones GR, Mills GA, Mermillod-Blondin F, Vulliet E (2020). Calibration and field application of an innovative passive sampler for monitoring groundwater quality. Talanta.

[CR89] Popek E (2018) Sampling and analysis of environmental chemical pollutants: A complete guide, 2nd edn. Elsevier. https://www.sciencedirect.com/book/9780128032022/sampling-and-analysis-of-environmental-chemical-pollutants. Accessed May 2022

[CR90] Prommer H, Barry DA, Davis GB (1999). A one-dimensional reactive multi-component transport model for biodegradation of petroleum hydrocarbons in groundwater. Environ Model Softw.

[CR91] Puls RW, Barcelona MJ (1996) Low-flow (minimal drawdown) ground-water sampling procedures. US EPA Off Solid Waste Emerg Response EPA/540/S-95/504, p 12. https://www.epa.gov/remedytech/low-flow-minimal-drawdown-ground-water-sampling-procedures. Accessed May 2022

[CR92] Putzlocher R, Kueper BH, Reynolds DA (2006). Relative velocities of DNAPL and aqueous phase plume migration. J Contam Hydrol.

[CR93] Rayner JL, Slee D, Falvey S, Kookana R, Bekele E, Stevenson G, Lee A, Davis GB (2022). Laboratory batch representation of PFAS leaching from aged field soils: Intercomparison across new and standard approaches. Sci Tot Environ.

[CR94] Rayner JL, Bekele E, Donn M, Bastow T, Davis GB, Woodbury R, Furness A, Geste Y (2020) Australian case studies of light non-aqueous phase liquid (LNAPL) natural source zone depletion rates compared with conventional active recovery efforts, CRC CARE Technical Report no. 47, CRC for Contamination Assessment and Remediation of the Environment, Newcastle, Australia, p 263. https://crccare.com/technical-reports/

[CR95] Rivett MO, Wealthall GP, Dearden RA, McAlary TA (2011). Review of unsaturated-zone transport and attenuation of volatile organic compound (VOC) plumes leached from shallow source zones. J Contam Hydrol.

[CR96] Robbat A (1997) A guideline for dynamic workplans and field analytics: The keys to cost-effective site characterization and cleanup, environmental technology initiative. U.S. EPA, Washington, D.C. https://clu-in.org/download/char/dynwkpln.pdf. Accessed May 2022

[CR97] Ronen D, Magaritz M, Levy I (1986). A multi-layer sampler for the study of detailed hydrochemical profiles in groundwater. Water Res.

[CR98] Roy PS, Behera MD, Srivastav SK (2017). Satellite remote sensing: sensors, applications and techniques. Proc Natl Acad Sci India Sect A Phys Sci.

[CR99] Sakale G, Knite M, Teteris V, Tupureina V, Stepina S, Liepa E (2011). The investigation of sensing mechanism of ethanol vapour in polymer-nanostructured carbon composite. Cent Eur J Phys.

[CR100] Sego DC, Robertson PK, Sasitharan S, Kilpatrick BI, Pillai VS (1994). Ground freezing and sampling of foundation soils at Duncan dam. Can Geotech J.

[CR101] Sillmann J, Thorarinsdottir T, Keenlyside N, Schaller N, Alexander LV, Hegerl G, Seneviratne SI, Vautard R, Zhang X, Zwiers FW (2017). Understanding, modeling and predicting weather and climate extremes: Challenges and opportunities. Weather Clim Extremes.

[CR102] Smith JWN, Davis GB, DeVaull GE, Garg S, Newell CJ, Rivett MO (2022). Natural Source Zone Depletion (NSZD): From process understanding to effective implementation at LNAPL-impacted sites. Q J Eng Geol Hydrogeol.

[CR103] Soedergren A (1987). Solvent-filled dialysis membranes simulate uptake of pollutants by aquatic organisms. Environ Sci Technol.

[CR104] Sookhak Lari K, Rayner JL, Davis GB (2017). A computational assessment of representative sampling of soil gas using existing groundwater monitoring wells screened across the water table. J Haz Mater.

[CR105] Sookhak Lari K, Davis GB, Rayner JL, Bastow TP, Puzon GJ (2019). Natural source zone depletion of LNAPL: a critical review supporting modelling approaches. Water Res.

[CR106] Sookhak Lari K, Davis GB, Rayner JL (2022). Towards a digital twin for characterising natural source zone depletion: a feasibility study based on the Bemidji site. Water Res.

[CR107] Sookhak Lari K, Johnston CD, Davis GB (2016) Gasoline multi-phase and multi-component partitioning in the vadose zone: dynamics and risk longevity. Vadose Zone J 15(3). 10.2136/vzj2015.07.0098

[CR108] The Driller (2022) Drilling Through History. Web page: Drilling Through History | The Driller, accessed June 2022

[CR109] Theocharopoulos SP, Wagner G, Sprengart J, Mohr M-E, Desaules A, Muntau H, Christou M, Quevauviller P (2001). European soil sampling guidelines for soil pollution studies. Sci Tot Environ.

[CR110] UK EA (2022) Land contamination: technical guidance. United Kingdom Environment Agency. Land contamination: technical guidance - GOV.UK (www.gov.uk). Updated February 2022; Accessed June 2022

[CR111] US EPA (1997) Expedited site assessment tools for UST sites: A guide for regulators. EPA 510-B-97–001. https://www.epa.gov/ust/expedited-site-assessment-tools-underground-storage-tank-sites-guide-regulators. Accessed June 2022

[CR112] van Liedekerke M, Prokop G, Rabl-Berger S, Kibblewhite M, Louwagie G (2014) Progress in the Management of Contaminated Sites in Europe. EUR 26376. Joint Research Centre, Institute for Environment and Sustainability, Scientific and Technical Research series. Luxembourg: Publications Office of the European Union, p 64. https://op.europa.eu/en/publication-detail/-/publication/b217d1ca-df14-4866-8cab-addd22fb2184. Accessed May 2022

[CR113] van Rossum G (2003) An introduction to python. Revised and updated version 2.5. Published by Network Theory Ltd. 164. Web: An Introduction to Python by Guido van Rossum. Accessed May 2022

[CR114] Wagner GH (1962). Use of porous ceramic cups to sample soil water within the profile. Soil Sci.

[CR115] Wang YG, Li Q, Zhang WS, Hu S, Peng H (2021). The architecture and application of an automatic operational model system for basin scale water environment management and design making supporting. J Environ Manag.

[CR116] Zhang S, Cheng Q (2021). Data analysis and management system design of contaminated site based on intelligent data acquisition vehicle and 5G communication. Int J Commun Syst.

